# Chondrogenic Differentiation of Human Adipose-Derived Stem Cells: A New Path in Articular Cartilage Defect Management?

**DOI:** 10.1155/2014/740926

**Published:** 2014-06-12

**Authors:** Jan-Philipp Stromps, Nora Emilie Paul, Björn Rath, Mahtab Nourbakhsh, Jürgen Bernhagen, Norbert Pallua

**Affiliations:** ^1^Department of Plastic Surgery, Hand Surgery, Burn Center, University Hospital RWTH Aachen, Pauwelsstraße 30, 52074 Aachen, Germany; ^2^Institute of Biochemistry and Molecular Cell Biology, University Hospital RWTH Aachen, Pauwelsstraße 30, 52074 Aachen, Germany; ^3^Department of Orthopedic Surgery, University Hospital RWTH Aachen, Pauwelsstraße 30, 52074 Aachen, Germany

## Abstract

According to data published by the Centers for Disease Control and Prevention, over 6 million people undergo a variety of medical procedures for the repair of articular cartilage defects in the U.S. each year. Trauma, tumor, and age-related degeneration can cause major defects in articular cartilage, which has a poor intrinsic capacity for healing. Therefore, there is substantial interest in the development of novel cartilage tissue engineering strategies to restore articular cartilage defects to a normal or prediseased state. Special attention has been paid to the expansion of chondrocytes, which produce and maintain the cartilaginous matrix in healthy cartilage. This review summarizes the current efforts to generate chondrocytes from adipose-derived stem cells (ASCs) and provides an outlook on promising future strategies.

## 1. Introduction

The loss of cartilage tissue due to trauma, tumor, or age-related degeneration is generally associated with poor prognosis and symptoms that require long-term follow-up treatment and represents an ongoing clinical challenge in hand and orthopedic surgery. Due to the limited vascularization of cartilage tissue, chondrocytes* in vivo* have poor proliferative activity and regenerative capacity. This limitation often leads to the accelerated development of osteoarthritis or the remodeling of cartilage defects with fibrous or fibrocartilaginous tissue, which has decreased mechanical potential compared with hyaline cartilage.

Autologous chondrocyte transplantation (ACT) was the first chondrocyte tissue engineering technique to be applied in daily clinical practice, accomplished by Brittberg et al., in 1994 [1]. This technique consists of three main steps, including the isolation of chondrocytes from healthy cartilage tissue, chondrocyte cultivation or expansion* in vitro* over 2-3 weeks, and the reinjection of chondrocytes into the injured cartilage covered with a periosteal flap [1]. This method gained international acceptance within the orthopedic surgery field [2] and was further refined by adding biomaterials such as coated scaffolds, membranes, and different matrices [3, 4]. Despite this wide acceptance, several studies have revealed certain problems and limitations of ACT, including cell leakage, the requirement for high cell concentrations, and apoptosis of the reinjected chondrocytes. In addition, the major shortcomings of this procedure remain; that is, only smaller cartilage defects can be addressed and an adjunct-qualified laboratory unit within the surgical department is necessary [3, 5].

The use of embryonic stem cells (ESCs) has been suggested to obtain a high number of autologous chondrocytes. However, ethical concerns have limited the clinical application of ESCs. Recent advances in induced pluripotent stem cell (iPSC) research have clearly shown that differentiated somatic cells can be reprogrammed into a multipotent state. Since the first description of iPSCs in 2006 was by Yamanaka et al., this new field has grown continuously, and different experimental approaches to nuclear reprogramming, including nuclear transfer, cell fusion, and transcription-factor transduction, have been developed [6, 7]. Despite these achievements, the clinical use of iPSCs seems to be limited for the near future due to the controversies concerning the high risk of inducing teratomas and tumor growth [8, 9].

The potential of adipose tissue in regenerative medicine has been underestimated for a long time, being reduced to the simple function of energy storage. The first report on the application of fat tissue for autologous reconstruction was published by Neuber, who performed a lipofilling procedure in an infraorbital rim in 1893 [13]. Two years later, Czerny reported the use of a Leoma for breast reconstruction [14]. In 1987, Bircoll and Novack used a lipoaspirate for breast recontouring [15]. Since these early studies, numerous studies have confirmed the efficacy of the isolation and application of adult adipose-derived stem cells (ASCs) in reconstructive medicine [10, 16, 17]. Detailed protein expression analyses have revealed a significant level of growth factors and proliferation-modulating proteins in lipoaspirates, highlighting their exceptional regenerative potential and developmental plasticity [18]. In recent years, studies using ASCs for various applications in tissue engineering and biomedical research have become widespread [19].

Regarding* in vivo* studies, the use of bone marrow-derived mesenchymal stem cells (BMSCs) has proven to be an effective new treatment strategy for the repair of damaged cartilage in several animal models. Additionally, recent studies in rabbits compared mesenchymal stem cell lines from different sources with a focus on their chondrogenic potential and showed slightly better results for BMSCs; however, ASCs were also capable of substantial cartilage remodeling [20, 21]. These findings were confirmed by Jung et al., who were able to detect* de novo* cartilage formation* in vivo* by injecting ASCs in combination with fibrin glue subcutaneously in nude mice [22]. Since 2012, ADIPOA, a new EU-funded research project, has been testing the treatment of osteoarthritis using ASCs injected into the diseased joints to activate and enhance the self-regeneration of hyaline cartilage [23].

Therefore, reviews of the current concepts, such as the chondrogenic differentiation of ASCs via mechanical forces, as well as* in vivo* studies, are of great interest in this dynamic field of tissue engineering.

## 2. Adipose-Derived Stem Cells (ASCs)

Mesenchymal stem cells (MSCs) are multipotent mesoderm-derived progenitor cells that can be isolated from various human tissues, including bone marrow (BMSCs), umbilical cord blood (CBSCs), muscular tissue (MDSCs), and adipose tissue (ASCs). MSCs derived from human adipose tissue have been successfully differentiated into functional adult white or brown fat cells as well as neural, muscle, tendon, bone, or cartilage cells [10, 24, 25]. However, variations in tissue sources and conditions as well as isolation techniques have led to variability in the MSC-initiating populations.

To minimize MSC population variability, the initial population of cells and the differentiated progeny are defined by examining the expression of specific cell surface markers. In addition to different surface antigen profiles, the individual therapeutic capacities of MSC populations can also greatly differ. For instance, in the treatment of myocardial infarction, MSCs from discrete populations demonstrate different healing performances in cardiac regeneration [26] but possess nearly equal chondrogenic differentiation capacities* in vitro* and* in vivo* [20, 27]. Additional factors, such as age and sex, have marked effects on the proliferation and differentiation capacities of ASCs. For example, ASCs from elderly donors (>60 years of age) display lower proliferation rates and impaired osteogenic and chondrogenic differentiation, whereas adipogenic differentiation is independent of donor age [28]. The donor's gender must also be taken into consideration because muscle-derived stem cells from female donors demonstrate a higher potential for cartilage regeneration and repair [29]. The differentiation potential and mechanical properties of ASCs also decline with extended cell passaging [30]. Therefore, many protocols and tissue engineering strategies utilize cells between the second and fifth passages.

Compared with other MSCs, a shift to utilizing ASCs has recently taken place because of their better bioavailability and easier harvesting by liposuction.

### 2.1. Harvesting Techniques for ASCs

In general, two principal techniques are used to harvest adipose tissue from the human body: plastic section and liposuction. For liposuction, different tumescent solutions have been developed in recent decades. Among the pioneers in modern liposuction, Illouz applied a technique initially introduced by Fischer, who used blunt cannulas for suction of subcutaneous fat tissue [31, 32]. Later, Coleman [16, 33] examined the regenerative potential of the lipoaspirate for reconstructive approaches. Liposuction and lipofilling are considered to be safe and minimally invasive techniques, but there are still some risks associated with these procedures. For instance, side effects in the early postoperative period, such as swelling, redness, itching, bruising, and, less frequently, hematoma formation, are common.

The Coleman technique is still the most frequently used harvesting protocol; however, other techniques, such as microharvesting using smaller cannulas for suction, as proposed by Nguyen et al., have also gained popularity [34]. Many studies have focused on comparisons among different techniques and the impact of these methods on the properties of the isolated ASCs [35]. Recently, a comparative clinical trial comparing the Magalon and the Coleman techniques revealed that microharvesting may be more suitable for tissue engineering and regenerative approaches because this technique results in ASCs with greater viability and migration potential [36].

The medical device industry also focuses on this issue by providing systems that promise increased cell viability and ASC enrichment, such as Celution, Cytori Lipobank (Cytori Therapeutics Inc., San Diego, CA, USA), Adivive (Palomar Medical Technologies Inc., Burlington, MA, USA), and a constantly growing number of similar products.

Although the Coleman technique is still the international standard for harvesting adipose tissue, a future shift to other techniques and further refinements seems likely.

### 2.2. Isolation of ASCs from Lipoaspirate

**Figure 1:**
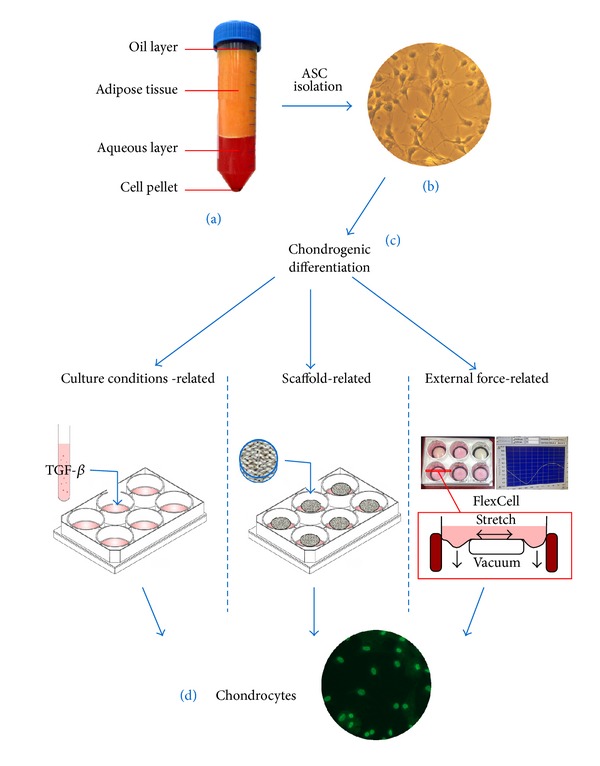
Concepts of the chondrogenic differentiation of ASCs: (a) human lipoaspirate after centrifugation, (b) isolated ASCs* in vitro*, (c) different induction methods for chondrogenic differentiation, and (d) SOX-9 immunostaining for chondrogenic detection.

The isolation of ASCs from freshly harvested lipoaspirate relies on well-established protocols [37]. After lipoaspirate collection, the adipose tissue is separated from the tumescent solution, oil, serum, cell debris, and blood by centrifugation. Coleman suggests centrifugation at 3,000 revolutions per minute for three minutes, which is often immediately performed in the operating theater. The resulting upper oil phase, the cellular debris, and the erythrocyte sediment are discarded (Figure 1(a)). The remaining adipose tissue is incubated with collagenase for up to one hour. Undigested structures are removed by subsequent filtration through a 250 *μ*m nylon mesh. The filtrate is then centrifuged to separate the stromal vascular fraction (SVF) from free lipids and mature adipocytes. After several washing steps and an optional erythrocyte lysis, the SVF cells are resuspended in proliferation medium and cultured for 24 hours to produce adherent cells.

The weakness of this procedure is that there are occasionally a low number of viable cells due to excessive lysis. Therefore, many variations have been introduced into the original protocol in recent years, and most laboratories apply slightly modified protocols, which have been established based on practical experience.

## 3. Concepts of Chondrogenic Differentiation

A variety of differentiation protocols have been published to achieve the chondrogenic differentiation of ASCs (Figure 1). These techniques are based on essentially dissimilar concepts and thus attain different levels of success. Concerning the time required, however, most differentiation protocols can be accomplished within 21 to 28 days.

### 3.1. Concepts Related to Culture Conditions

The chondrogenic differentiation of ASCs* in vitro* can be induced by adding various supplements and growth factors to the basic medium. In this regard, factors, such as transforming growth factors beta 1 and 3 (TGF-*β* 1, TGF-*β* 3), bone morphogenetic protein 4 (BMP 4), sex determining region Y box 9 (SOX 9), and basic fibroblast growth factor (bFGF), alone or in combination, have high chondrogenic potential. Certain animal models have demonstrated the importance of FGF 2 signaling activation for the induction of cartilaginous repair in full-thickness articular cartilage defects [38]. Moreover, high concentrations of bFGF and TGF-*β* 1 in human wound fluids have been demonstrated to be important in the healing process and can also be found in lipoaspirates [39].

The most commonly used protocols for the chondrogenic differentiation of ASCs involve the supplementation of basic medium (Dulbecco's modified Eagle's medium + 1% fetal calf or bovine serum and dexamethasone) with ascorbate-2-phosphate (50 nM ASAP), TGF-*β* 1 (10 ng/mL), and insulin (6.25 *μ*g/mL).

The industry currently offers a large variety of ready-to-use chondrogenesis supplements for MSCs, such as OriCell (Cyagen, GUXMX-90041, Santa Clara, CA, USA), PromoCell C-28013 (PromoCell, Heidelberg, Germany), and StemPro A10071-01 chondrogenesis differentiation kits (Gibco/Life Technologies, Darmstadt, Germany).  Table 1 provides an overview of different media for the chondrogenic differentiation of ASCs.

In general, cartilage and the resident chondrocytes are exposed to low oxygen tension ranging from 2 to 7% saturation [40]. Several studies have reported that this low oxygen tension enhances the chondrogenic differentiation of BMSCs in the presence of induction medium [41, 42]. Most importantly, oxygen deprivation (1% oxygen) enhances ASC proliferation, and 5% oxygen promotes the chondrogenesis of ASCs* in vitro* [43, 44]. These data emphasize the importance of oxygen concentration during stem cell growth and differentiation. In contrast, hypoxia was shown to induce the macrophage inhibitory factor MIF, which has been recently found to be involved in a degenerative process of the cartilage end plates [45–47]. Thus, it remains unknown whether chondrogenic commitment under hypoxic conditions positively affects the formation of cartilaginous tissue* in vivo*.

### 3.2. Scaffold-Related Concepts and Three-Dimensional Culturing

*In vitro*, ASCs tend to grow as a monolayer in cell culture and avoid cell-cell contact by growth inhibition. However, excessive cell accumulation, as occurring in high-density micropellets, is a fundamental prerequisite for chondrogenic differentiation. In recent years, three-dimensional (3D) constructs, such as scaffolds, various hydrogels, alginate gels, and matrices, have been developed to overcome growth inhibition [12]. These 3D carriers mimic the physiological milieu. Similarly, scaffolds covered with different chemotactic agents, as well as matrices of varying stiffness values, have been designed to achieve directional migration and stable cell cultures. In 2007, Xu et al. were among the first groups to focus on the mechanical properties of chondrocyte differentiation with a 3D mass model [48].

### 3.3. Chondrogenic Differentiation via Mechanical Forces

The induction of stem cell differentiation by applying mechanical forces is an innovative concept in artificial tissue generation. It has been established that the cytoskeleton interprets and responds differentially to mechanical forces from the microenvironment [30]. In this context, cellular actin filaments were shown to be a key initial regulator of cell morphology in response to extracellular mechanical forces [30].

*In vitro*, different strategies can be used to apply mechanical forces to cells. The FlexCell system (FlexCell Tension System FX-5000T, Dunn Labortechnik GmbH, Asbach, Germany) is an up-to-date technique that is based on seeding the cells on a silicone membrane that can be stretched or flexed in a static or cyclic mode using vacuum pressure. Other strategies focus on applying pressure to cell cultures with static or dynamic stamps or centrifugal force.

Although preliminary results show a high potential and promising future for mechanical chondrogenic differentiation in tissue engineering, the underlying molecular mechanisms have not yet been extensively studied in detail.

## 4. Detection of Chondrogenic Differentiation

Numerous methods have been applied in the past to monitor and evaluate the process of chondrogenic differentiation [49]. These methods range from lineage-specific immunological or histological assays to the direct detection of chondrocyte-specific extracellular matrix (ECM) protein expression, such as different collagen types (COL I, II, IV, IX, X, and XI), keratin sulfate, and chondroitin sulfate [12]. Additionally, real-time PCR, western blot analysis, ELISA, and RNA microarray analysis are widely used. Less laborious methods have also been described based on the detection of cartilage-like matrix production, such as the staining of sulfated proteoglycan-rich matrix using alcian blue, toluidine blue, or safranin-O staining. These methods represent an alternative to immunostaining for the presence of collagen type I (COL I), COL II, and COL X [49, 50]. In addition, the differentiation of ASCs to chondrocytes can be determined by genomic analysis, emphasizing the expression of COL II, COL X, and the genes for aggrecan, decorin, and biglycan, which are all genes specific for chondrocyte cell lines [12] (Table 2).

## 5. Conclusions and Outlook

Lipofilling is the mostly commonly used procedure for the volumetric correction of depressed scars, flap remodeling, breast reconstruction, or the treatment of contour deformities. Human ASCs have good potential to differentiate into cartilaginous tissues* in vitro* and* in vivo*. The differentiation of these cells can be induced by various stimuli.

Taken as a whole, ASCs represent a very promising resource for further research in cartilaginous tissue engineering due to the increased bioavailability and easier harvesting of ASCs compared with other mesenchymal stem cells. Nevertheless, the chondrogenic differentiation of human adipose-derived stem cells is still a dynamic field.

An ideal technique would combine liposuction with the intraoperative isolation of ASCs and the enrichment of these cells with chondrogenic factors, followed by the direct injection of this cocktail into osteoarthritic joints. Many researchers worldwide are working on attaining these ideal conditions, but numerous challenges remain, such as clinical safety issues and the issue of immune responses to an allogeneic transplantation.

## Figures and Tables

**Table 1:** Media for the chondrogenic differentiation of ASCs.

	Basal medium	Supplements
(1)	DMEM + 1% FCS	(i) TGF-*β*1 (10 ng/mL)
		(ii) ASAP (50 nM)
		(iii) Insulin (6.25 *μ*g/mL)

(2)	DMEM	(i) TGF-*β*3
		(ii) Albumin (1.25 *μ*g/mL)
		(iii) Dexamethasone (10–7 M)
		(iv) Ascorbic acid
		(v) Transferrin (6.25 *μ*g/mL)
		(vi) Insulin (6.25 *μ*g/mL)

(3)	OriCell	(i) TGF-*β*3
		(ii) Dexamethasone
		(iii) Ascorbic acid
		(iv) ITS cell culture supplement
		(v) Sodium pyruvate
		(vi) Proline

According to (1) Zuk et al., [10] (2) Baptista et al., [11] (3) OriCell (Cyagen, GUXMX-90041, Santa Clara, CA, USA).

ASAP: ascorbate-2-phosphate; DMEM: Dulbecco's modified Eagle's medium; FCS: fetal calf serum; TGF: transforming growth factor.

**Table 2:** Expressed chondrogenic genes that are detectable at the different stages of differentiation over time (0 to 21 days) [12].

Chondrogenic differentiation	Expressed genes
Stage I	Collagen I, collagen VI
	SOX 4
	BMP 2

Stage II	COMP
	HAPLN1
	Collagen XI
	SOX 9

Stage III	Matrilin 3
	Indian hedgehog
	Homeobox 7
	Chondroadherin
	WNT 11

Stage IV	Aggrecan
	Alkaline phosphatase
	Collagen II, collagen IX, and collagen X
	Fibromodulin
	Osteocalcin
	PTHrP

## References

[1] Brittberg M, Lindahl A, Nilsson A, Ohlsson C, Isaksson O, Peterson L (1994). Treatment of deep cartilage defects in the knee with autologous chondrocyte transplantation. *The New England Journal of Medicine*.

[2] Micheli LJ, Browne JE, Erggelet C (2001). Autologous chondrocyte implantation of the knee: multicenter experience and minimum 3-year follow-up. *Clinical Journal of Sport Medicine*.

[3] Steinwachs M (2009). New technique for cell-seeded collagen-matrix-supported autologous chondrocyte transplantation. *Arthroscopy*.

[4] Tohyama H, Yasuda K, Minami A (2009). Atelocollagen-associated autologous chondrocyte implantation for the repair of chondral defects of the knee: a prospective multicenter clinical trial in Japan. *Journal of Orthopaedic Science*.

[5] Gille J, Ehlers E-M, Okroi M, Russlies M, Behrens P (2002). Apoptotic chondrocyte death in cell-matrix biocomposites used in autologous chondrocyte transplantation. *Annals of Anatomy*.

[6] Takahashi K, Yamanaka S (2006). Induction of pluripotent stem cells from mouse embryonic and adult fibroblast cultures by defined factors. *Cell*.

[7] Yamanaka S, Blau HM (2010). Nuclear reprogramming to a pluripotent state by three approaches. *Nature*.

[8] Lu Y, Xu D, Zhou J (2013). Differential responses to genotoxic agents between induced pluripotent stem cells and tumor cell lines. *Journal of Hematology & Oncology*.

[9] Yang J, Lam DH, Goh SS (2012). Tumor tropism of intravenously injected human-induced pluripotent stem cell-derived neural stem cells and their gene therapy application in a metastatic breast cancer model. *Stem Cells*.

[10] Zuk PA, Zhu M, Mizuno H (2001). Multilineage cells from human adipose tissue: implications for cell-based therapies. *Tissue Engineering*.

[11] Baptista LS, Silva KR, Pedrosa CS (2013). Bioengineered cartilage in a scaffold-free method by human cartilage-derived progenitor cells: a comparison with human adipose-derived mesenchymal stromal cells. *Artificial Organs*.

[12] Xu J, Wang W, Ludeman M (2008). Chondrogenic differentiation of human mesenchymal stem cells in three-dimensional alginate gels. *Tissue Engineering A*.

[13] Neuber GA (1893). Verfahren subkutaner Fettimplantation. *Verhandlungen der Deutschen Gesellschaft für Chirurgie*.

[14] Czerny V (1895). Plastischer Ersatz der Brustdrüse durch ein Lipom. Plastische Operationen. *Verhandlungen der Deutschen Gesellschaft für Chirurgie*.

[15] Bircoll M, Novack BH (1987). Autologous fat transplantation employing liposuction techniques. *Annals of Plastic Surgery*.

[16] Coleman SR (1997). Facial recountouring with lipostructure. *Clinics in Plastic Surgery*.

[17] Gimble JM, Nuttall ME (2011). Adipose-derived stromal/stem cells (ASC) in regenerative medicine: pharmaceutical applications. *Current Pharmaceutical Design*.

[18] Pallua N, Pulsfort AK, Suschek C, Wolter TP (2009). Content of the growth factors bFGF, IGF-1, VEGF, and PDGF-BB in freshly harvested lipoaspirate after centrifugation and incubation. *Plastic and Reconstructive Surgery*.

[19] Wei Y, Sun X, Wang W, Hu Y (2007). Adipose-derived stem cells and chondrogenesis. *Cytotherapy*.

[20] Xie X, Wang Y, Zhao C (2012). Comparative evaluation of MSCs from bone marrow and adipose tissue seeded in PRP-derived scaffold for cartilage regeneration. *Biomaterials*.

[21] Li Q, Tang J, Wang R (2011). Comparing the chondrogenic potential in vivo of autogeneic mesenchymal stem cells derived from different tissues. *Artificial Cells, Blood Substitutes, and Biotechnology*.

[22] Jung S-N, Rhie JW, Kwon H (2010). In vivo cartilage formation using chondrogenic-differentiated human adipose-derived mesenchymal stem cells mixed with fibrin glue. *Journal of Craniofacial Surgery*.

[23] Jorgensen C (2011). ADIPOA: cell therapy with stromal adipocytes cells. *Revue de Medecine Interne*.

[24] Gimble JM (2012). Stem cells bleed into brown fat. *Cell Metabolism*.

[25] Heydarkhan-Hagvall S, Schenke-Layland K, Yang JQ (2008). Human adipose stem cells: a potential cell source for cardiovascular tissue engineering. *Cells Tissues Organs*.

[26] Gaebel R, Furlani D, Sorg H (2011). Cell origin of human mesenchymal stem cells determines a different healing performance in cardiac regeneration. *PLoS ONE*.

[27] Berg LC, Koch TG, Heerkens T, Besonov K, Thomsen PD, Betts DH (2009). Chondrogenic potential of mesenchymal stromal cells derived from equine bone marrow and umbilical cord blood. *Veterinary and Comparative Orthopaedics and Traumatology*.

[28] Choudhery MS, Badowski M, Muise A, Pierce J, Harris DT Donor age negatively impacts adipose tissue-derived mesenchymal stem cell expansion and differentiation. *Journal of Translational Medicine*.

[29] Matsumoto T, Kubo S, Meszaros LB (2008). The influence of sex on the chondrogenic potential of muscle-derived stem cells implications for cartilage regeneration and repair. *Arthritis and Rheumatism*.

[30] Gonzalez-Cruz RD, Darling EM (2013). Adipose-derived stem cell fate is predicted by cellular mechanical properties. *Adipocyte*.

[31] Fischer G (1990). Liposculpture: the “correct” history of liposuction—part I. *Journal of Dermatologic Surgery and Oncology*.

[32] Illouz YG (1986). The fat cell “graft”: a new technique to fill depressions. *Plastic and reconstructive surgery*.

[33] Coleman SR (1995). Long-term survival of fat transplants: controlled demonstrations. *Aesthetic Plastic Surgery*.

[34] Nguyen PSA, Desouches C, Gay AM, Hautier A, Magalon G (2012). Development of micro-injection as an innovative autologous fat graft technique: the use of adipose tissue as dermal filler. *Journal of Plastic, Reconstructive and Aesthetic Surgery*.

[35] Fisher C, Grahovac TL, Schafer ME, Shippert RD, Marra KG, Rubin JP (2013). Comparison of harvest and processing techniques for fat grafting and adipose stem cell isolation. *Plastic and Reconstructive Surgery*.

[36] Alharbi Z, Opländer C, Almakadi S, Fritz A, Vogt M, Pallua N (2013). Conventional vs. micro-fat harvesting: how fat harvesting technique affects tissue-engineering approaches using adipose tissue-derived stem/stromal cells. *Journal of Plastic, Reconstructive and Aesthetic Surgery*.

[37] Bunnell BA, Flaat M, Gagliardi C, Patel B, Ripoll C (2008). Adipose-derived stem cells: isolation, expansion and differentiation. *Methods*.

[38] Hiraki Y, Shukunami C, Iyama K, Mizuta H (2001). Differentiation of chondrogenic precursor cells during the regeneration of articular cartilage. *Osteoarthritis and Cartilage*.

[39] Pallua N, Ulrich D (2003). Expression of basic fibroblast growth factor and transforming growth factor-beta 1 in patients with fasciocutaneous and muscle flaps. *Plastic and Reconstructive Surgery*.

[40] Zhou S, Cui Z, Urban JPG (2004). Factors influencing the oxygen concentration gradient from the synovial surface of articular cartilage to the cartilage-bone interface: a modeling study. *Arthritis and Rheumatism*.

[41] Amarilio R, Viukov SV, Sharir A, Eshkar-Oren I, Johnson RS, Zelzer E (2007). HIF1*α* regulation of Sox9 in necessary to maintain differentiation of hypoxic prechondrogenic cells during early skeletogenesis. *Development*.

[42] Markway BD, Tan G-K, Brooke G, Hudson JE, Cooper-White JJ, Doran MR (2010). Enhanced chondrogenic differentiation of human bone marrow-derived mesenchymal stem cells in low oxygen environment micropellet cultures. *Cell Transplantation*.

[43] Merceron C, Vinatier C, Portron S (2010). Differential effects of hypoxia on osteochondrogenic potential of human adipose-derived stem cells. *American Journal of Physiology—Cell Physiology*.

[44] Weijers EM, van den Broek LJ, Waaijman T, van Hinsbergh VWM, Gibbs S, Koolwijk P (2011). The influence of hypoxia and fibrinogen variants on the expansion and differentiation of adipose tissue-derived mesenchymal stem cells. *Tissue Engineering A*.

[45] Pallua N, Serin M, Wolter TP (2014). Characterisation of angiogenetic growth factor production in adipose tissue-derived mesenchymal cells. *Journal of Plastic Surgery and Hand Surgery*.

[46] Simons D, Grieb G, Hristov M (2011). Hypoxia-induced endothelial secretion of macrophage migration inhibitory factor and role in endothelial progenitor cell recruitment. *Journal of Cellular and Molecular Medicine*.

[47] Skurk T, Herder C, Kräft I, Müller-Scholze S, Hauner H, Kolb H (2005). Production and release of macrophage migration inhibitory factor from human adipocytes. *Endocrinology*.

[48] Xu Y, Balooch G, Chiou M, Bekerman E, Ritchie RO, Longaker MT (2007). Analysis of the material properties of early chondrogenic differentiated adipose-derived stromal cells (ASC) using an in vitro three-dimensional micromass culture system. *Biochemical and Biophysical Research Communications*.

[49] Steinert AF, Palmer GD, Pilapil C, Nöth U, Evans CH, Ghivizzani SC (2009). Enhanced in vitro chondrogenesis of primary mesenchymal stem cells by combined gene transfer. *Tissue Engineering A*.

[50] Meretoja VV, Dahlin RL, Kasper FK, Mikos AG (2012). Enhanced chondrogenesis in co-cultures with articular chondrocytes and mesenchymal stem cells. *Biomaterials*.

